# All Roads Lead to Rome: Diverse Etiologies of Tricuspid Regurgitation Create a Predictable Constellation of Right Ventricular Shape Changes

**DOI:** 10.3389/fphys.2022.908552

**Published:** 2022-06-02

**Authors:** Benjamin A. Orkild, Brian Zenger, Krithika Iyer, Lindsay C. Rupp, Majd M Ibrahim, Atefeh G. Khashani, Maura D. Perez, Markus D. Foote, Jake A. Bergquist, Alan K. Morris, Jiwon J. Kim, Benjamin A. Steinberg, Craig Selzman, Mark B. Ratcliffe, Rob S. MacLeod, Shireen Elhabian, Ashley E. Morgan

**Affiliations:** ^1^ Scientific Computing and Imaging Institute, University of Utah, Salt Lake City, UT, United States; ^2^ Department of Biomedical Engineering, University of Utah, Salt Lake City, UT, United States; ^3^ School of Computing, University of Utah, Salt Lake City, UT, United States; ^4^ Weill-Cornell Medical College, Division of Cardiology, New York, NY, United States; ^5^ Division of Cardiovascular Medicine, University of Utah, Salt Lake City, UT, United States; ^6^ Division of Cardiothoracic Surgery, University of Utah, Salt Lake City, UT, United States; ^7^ Department of Surgery, The San Francisco VA Medical Center, University of California, San Francisco, San Francisco, CA, United States; ^8^ St. Luke’s Medical Center Cardiothoracic and Vascular Surgery, Boise, ID, United States

**Keywords:** pulmonary hypertension, tricuspid regurgitation, statistical shape modeling, cardiac MRI, particle-based shape modeling, principal component analysis, congestive heart failure, valvular heart disease

## Abstract

**Introduction:** Myriad disorders cause right ventricular (RV) dilation and lead to tricuspid regurgitation (TR). Because the thin-walled, flexible RV is mechanically coupled to the pulmonary circulation and the left ventricular septum, it distorts with any disturbance in the cardiopulmonary system. TR, therefore, can result from pulmonary hypertension, left heart failure, or intrinsic RV dysfunction; but once it occurs, TR initiates a cycle of worsening RV volume overload, potentially progressing to right heart failure. Characteristic three-dimensional RV shape-changes from this process, and changes particular to individual TR causes, have not been defined in detail.

**Methods:** Cardiac MRI was obtained in 6 healthy volunteers, 41 patients with ≥ moderate TR, and 31 control patients with cardiac disease without TR. The mean shape of each group was constructed using a three-dimensional statistical shape model via the particle-based shape modeling approach. Changes in shape were examined across pulmonary hypertension and congestive heart failure subgroups using principal component analysis (PCA). A logistic regression approach based on these PCA modes identified patients with TR using RV shape alone.

**Results:** Mean RV shape in patients with TR exhibited free wall bulging, narrowing of the base, and blunting of the RV apex compared to controls (*p* < 0.05). Using four primary PCA modes, a logistic regression algorithm identified patients with TR correctly with 82% recall and 87% precision. In patients with pulmonary hypertension without TR, RV shape was narrower and more streamlined than in healthy volunteers. However, in RVs with TR and pulmonary hypertension, overall RV shape continued to demonstrate the free wall bulging characteristic of TR. In the subgroup of patients with congestive heart failure without TR, this intermediate state of RV muscular hypertrophy was not present.

**Conclusion:** The multiple causes of TR examined in this study changed RV shape in similar ways. Logistic regression classification based on these shape changes reliably identified patients with TR regardless of etiology. Furthermore, pulmonary hypertension without TR had unique shape features, described here as the “well compensated” RV. These results suggest shape modeling as a promising tool for defining severity of RV disease and risk of decompensation, particularly in patients with pulmonary hypertension.

## 1 Introduction

The tricuspid valve sits between the right atrium and the right ventricle, anchored to the walls of the right heart. In the healthy heart, the tricuspid valve preserves one-way blood flow, preventing blood from returning to the right atrium during RV contraction. Many diseases disturb tricuspid valve function, including direct valve injury during pacemaker implantation, dilation of the RV as it faces high resistance to flow in pulmonary hypertension or left ventricular failure, and dilation of the right atrium in atrial fibrillation (Prins et al., 2019). Any disorder rendering the tricuspid valve leaflets unable to fully close allows retrograde flow of blood back into the right atrium, also known as tricuspid regurgitation (TR). As the right atrium dilates in response to the excess blood volume arising from TR, the supporting structures of the tricuspid valve also dilate, worsening the TR. If left untreated, the end result of this cycle is right heart failure and death (Cameli et al., 2013; Dawes et al., 2017; Subbotina et al., 2017; Prins et al., 2019).

**FIGURE 1:**
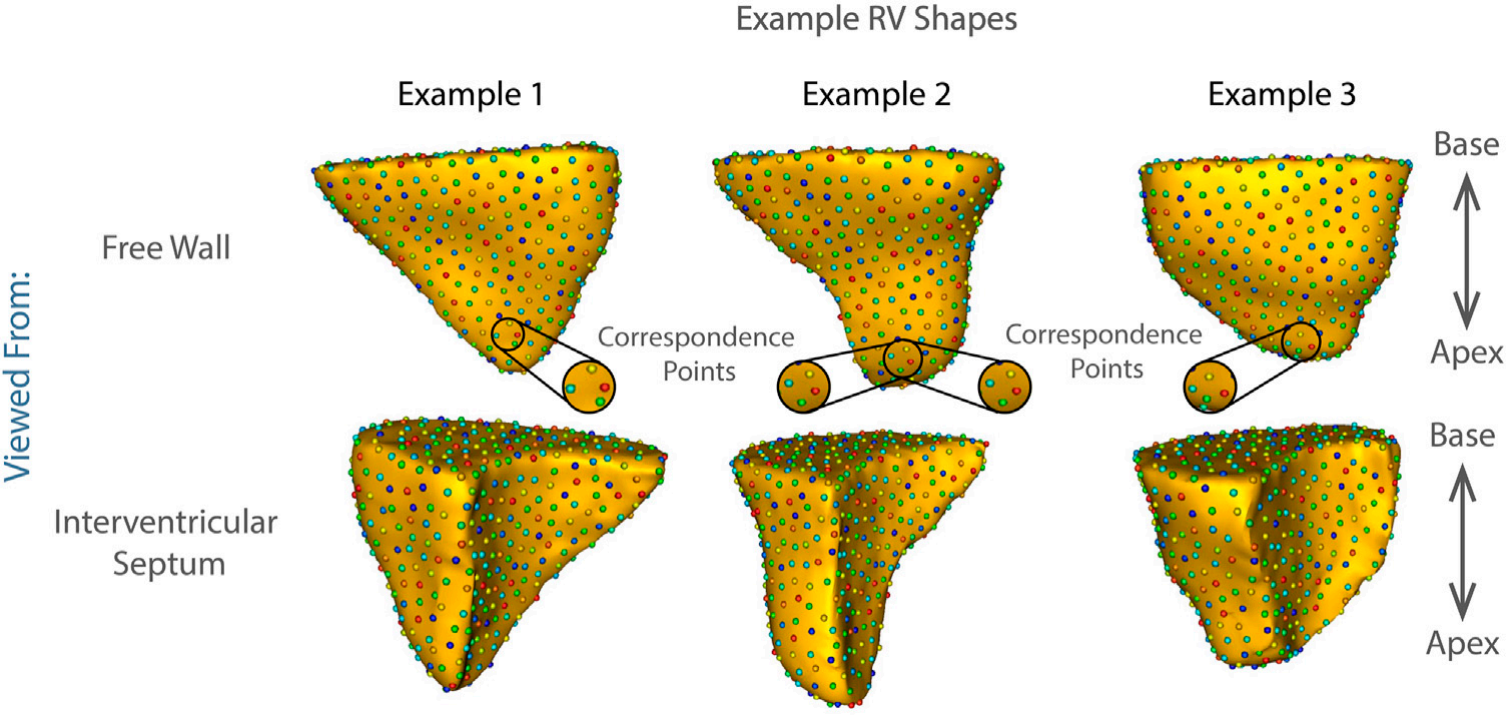
Examples of shape correspondence models of right ventricular shape obtained using ShapeWorks. The colored spheres on the surface of each RV are the optimized correspondence points/corresponding landmarks that fully parameterize the right ventricular shape based on the population data (see Methods: Statistical Shape Model Construction). The three examples showcase the complexity of the RV shapes in the cohort.

An estimated 1.6 million Americans currently live with moderate or greater TR (Stuge and Liddicoat, 2006). The diagnosis of TR alone places them at a 2–3 fold increased risk of death and a 3–4 fold increased risk of congestive heart failure, compared to age-matched individuals without TR (Topilsky et al., 2019; Singh et al., 1999). TR can be treatable with surgical valve repair or replacement, and with therapy targeted at the culprit cardiac disease, but morbidity and mortality of treatment rise with increasing RV dysfunction (Subbotina et al., 2017; Topilsky et al., 2019; LaPar et al., 2018). As the RV deteriorates in response to TR, it changes shape. The shape of the healthy RV is complex, and cannot be modeled as a simple shape such as a sphere, ovoid or cone. (Figure 1). Changes in RV geometry with disease have therefore been historically difficult to quantify. Previous studies relied on descriptions such as increasing resemblance to a sphere, or used global shape descriptors such as wall curvature (Morgan et al., 2020a; Addetia et al., 2018). With advances in cardiac imaging and statistical shape modeling, high resolution detailed RV shape analysis has become a promising avenue for identifying the fine details of RV shape change specific to particular pathologies (Figure 1).. (Marcu et al., 2006; Kochav et al., 2015; Farrar et al., 2016; Dawes et al., 2017; Mauger et al., 2019)

In this study, we sought to:

1) Develop a population-level anatomical description of RV shape, directly from detailed three-dimensional (3D) models of RVs generated from cardiac MRI.
2) Discover statistically significant group differences.
3) Classify the RV shape changes characteristic of TR.
4) Examine whether those changes are similar in various TR etiologies.

To accomplish these aims we obtained cardiac MRI in three groups: healthy volunteers; patients with diverse cardiac diseases resulting in moderate or greater TR; and a matched cohort of patients with cardiac disease without TR. We then employed a particle-based shape modeling tool, the open-source software package, ShapeWorks, which provides a computational approach to automatically parse shape into population-level numerical representations (Cates et al., 2017). Using this approach, we identified primary modes of shape variation between patients with and without TR, which were then applied using a logistic regression algorithm with automated feature selection to a testing subset to identify patients with TR based on RV shape alone.

In our recent publication, we used this technique to compare the RV shape of patients with TR to those of healthy volunteers (Morgan et al., 2020b). The current study expands significantly on that technique, as follows.

1) Here we included a matched cohort of patients with cardiac disease without TR, allowing analysis of progression of RV shape along a spectrum from complete health to significant impairment.
2) Beyond identifying changes in TR compared to healthy patients, here we also highlighted unique changes occurring in the specific disease states of pulmonary hypertension and congestive heart failure (CHF).
3) We increased the number of cases and improved the statistical power of our study. To mitigate the bias of the classification model due to the imbalanced dataset, we performed minority upsampling and increased the predictive performance of the model.
4) To gain insight into the geometric differences that are most statistically significant between subgroups, we performed linear discrimination of variation using the mean shape of the groups and mapping the high dimensional differences to a single scalar value.

We propose that these techniques will form the foundation of future predictive tools, with the potential to identify patients at risk for RV dysfunction in TR before their disease progresses.

## 2 Materials and Methods

### 2.1 Patient Selection

This study included three groups of patients:

1) Patients with moderate or greater TR
2) Comorbidity-matched controls with cardiac disease but without TR
3) Healthy volunteers without cardiac disease or risk factors

Groups 1 & 2 were identified retrospectively using the University of Utah medical data warehouse and a combination of procedure (cardiac MRI) and diagnosis (TR) codes. Each patient chart was manually reviewed to verify TR and sort patients by TR grade. This study was declared to be of minimal risk and granted exemption by the University of Utah IRB. Healthy volunteer images were obtained during a previous study at Weill-Cornell Medical College, after IRB approval (Morgan et al., 2018).

All MRI images were reviewed for quality, as well as for grade of TR and to classify ventricular volumes, by a board-certified cardiologist with additional certification in cardiac MRI (M Ibrahim). Images without clearly defined RV endocardial borders, or which did not include the entirety of the RV from tricuspid valve to apex, were excluded from analysis. TR grade was classified based on MRI regurgitant volume, or echocardiographic criteria if an echo had been obtained within 30 days of the MRI (Zhan et al., 2020).

### 2.2 Image Processing Pipeline

**FIGURE 2:**
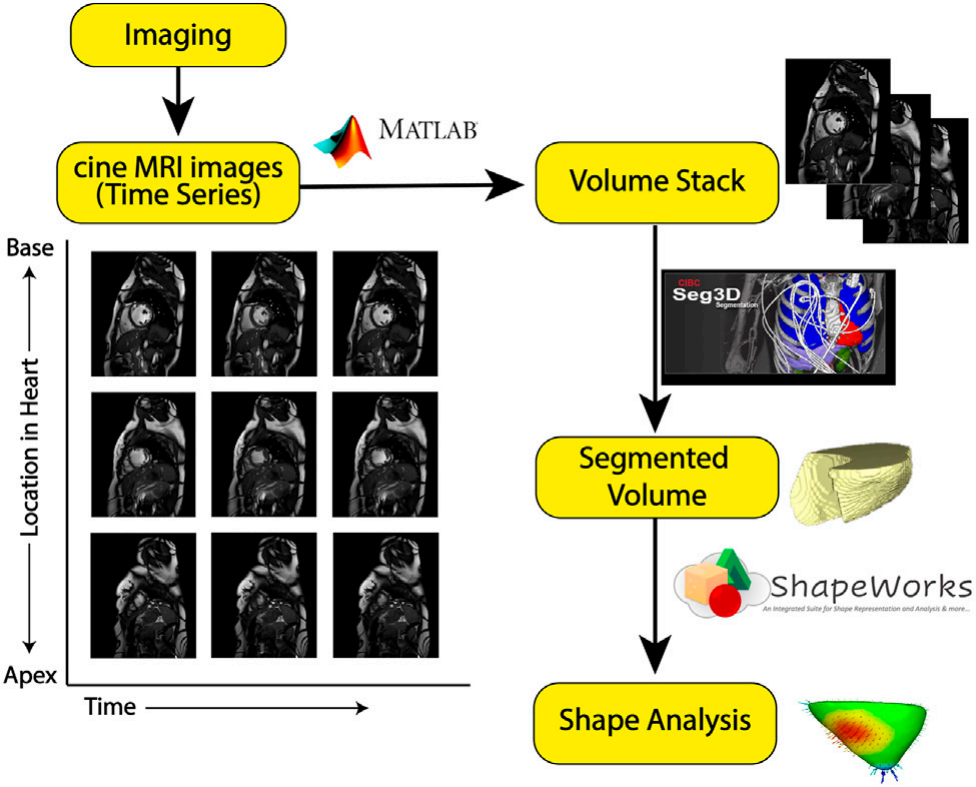
Image processing pipeline: The yellow boxes represent each step in the processing pipeline. The symbols along the arrows show the software tool used to go from one step to another. From cine-MRI images, volume stacks were created and segmented using Seg3D, then uploaded to ShapeWorks for particle-based shape modeling.

The overall method of image processing is depicted in Figure 2.

#### 2.2.1 MRI Imaging

MRI was performed on a 3T Prisma scanner (Siemens Healthineers, Erlangen Germany) using body and spine array coils. Cine MRI images were acquired as a stack of 12–16 short axis slices covering the RV from above the tricuspid valve to beyond the cardiac apex. Typical scan parameters for Cine MRI were true-FISP pulse sequence with TE/TR = 1.5/3.3 ms, flip angle (FA) = 50°, 6 mm slice thickness, spatial resolution of 1.25 × 1.56 mm, and temporal resolution of 23.4 ms.

#### 2.2.2 CINE MRI to Volume Stack

To create three-dimensional models of the RV at end-diastole, a volume stack was created from a series of CINE MRI scans. CINE MRI time stacks capture one short axis slice of the heart through time (multiple heart beats). From each CINE short axis time stack, an image of the heart at end diastole was extracted to create a volume image stack. End-diastole was manually identified as the time point immediately after tricuspid valve closure. Image extraction was performed using a custom-built MATLAB image processing tool, available at https://github.com/borkild/CINEtoVolume/releases/tag/1.0.

#### 2.2.3 Segmentation Creation and Processing

From the volume stacks, RV segmentations were created using the open-source Seg3D software (SCI Institute, University of Utah, SLC UT). RV endocardial segmentations were semi-automatically created using the “implicit model” tool. Implicit model seed points were placed along the endocardial border at each slice of the volume stack. Prior to running the implicit model tool, the image was upsampled in the *z*-direction using a box interpolation. This upsampled segmentation was then manually edited to remove artifacts or errors. The final segmentation was exported as a binary mask volume for further analysis.

Prior to shape analysis, segmentations were pre-processed using ShapeWorks tools, wherein the segmentations were isotropically resampled and rigidly aligned to have identical dimensions and centroids. Segmentations were then converted to distance transforms for shape analysis.

### 2.3 Shape Modeling Workflow

Statistical shape modeling (SSM) is a valuable and powerful tool to generate a detailed representation of complex anatomy that enables quantitative analysis and the comparison of shapes and their variations. The steps involved in shape modeling were:

1) Statistical shape-model construction with ShapeWorks
2) Modeling shape variation with the method of Principal Component Analysis (PCA)

To study the differences between controls and pathology subjects, we have designed downstream classification tasks that help us verify if the shape model obtained contains clinically relevant morphological features that capture the population level variability. Workflows for downstream classification tasks included:

1) Train-test split and upsampling of training data
2) Feature selection using lasso regression and classification

Analyzing specific pathology characteristics involved:

1) Linear discriminant analysis
2) Determining statistical significance of shape differences

Each of the workflow steps is explained in detail in the following section.

#### 2.3.1 Statistical Shape Model Construction

Shape modeling was performed using the open-source ShapeWorks (http://sciinstitute.github.io/ShapeWorks/) software. The methodology of ShapeWorks has been described previously (Cates et al., 2017). Briefly, the particle-based shape model of a shape sample represents its segmentation using a densely ordered set of computationally derived landmarks automatically placed on consistent 3D locations across the entire shape cohort. Therefore, such landmarks provide 3D correspondence points across the population, allowing for comparison between individual and group shapes and computation of statistical differences. In this application, 512 particles were distributed across the processed RV endocardial shapes using a gradient-descent optimization strategy. Particle placement was further optimized by minimizing the cost function associated with individual shapes compared to the overall shape population. This pipeline created a uniform distribution of particles that adequately represented each shape across each RV surface. From these particles, mean shapes and differences were computed.

#### 2.3.2 Modeling Shape Variation With Principal Component Analysis

Principal component analysis (PCA) was used to reduce the correspondence data to a smaller set of linearly uncorrelated components, determining the number of modes explaining significant shape variation. We mapped each RV shape to its respective PCA loading vector.

#### 2.3.3 Training and Testing

PCA loadings of RV shapes were divided into training and testing sets, using train_test_split in sklearn (scikit-learn.org) (Pedregosa et al., 2011). The training set comprised 80% of the population and the testing set 20%. We then upsampled the training data to increase the statistical power, minimizing the difference between group sizes to construct balanced cohorts. For this, we employed a version of the Synthetic Minority Oversampling Technique (SMOTE), known as Borderline-SMOTE (Han et al., 2005). This technique generates additional synthetic samples from the easily misclassified borderline region—the shapes between shapes definitely belonging to the patient set and those definitely belonging to the control set. In our dataset, the two minority groups were 1) patients with TR and 2) healthy controls; compared to a majority group of controls with comorbid conditions. Using Borderline-SMOTE, representative RV shapes of the minority groups were selected, and the shapes of their nearest neighbors identified. If those shapes belonged entirely to the same set as the selected shape, they were not selected for upsampling. However, if the shapes of its nearest neighbors belonged to the opposite group (e.g., patient, if the original is a healthy volunteer), it is considered to exist on the borderline, and it is upsampled by generating synthetic samples along the line of transformation from the original to the nearest neighbor shape. This process was repeated until the minority classes had the same proportion as the majority class.

#### 2.3.4 Feature Selection

We determined the number of PCA components required to explain 99% of the data variation, as follows: Assume *s*_*i*_ is the PCA loading vector for shape *i* and *y*_*i*_ is its corresponding label. We set *y*_*i*_ equal to 1 for patients and 0 for control subjects. Our objective was to identify the principal components (*i.e.,* shape-based parameters) that were most predictive of the patient group. In this regard, we solved the lasso regression for 1000 random subsets of data, and accordingly found a weight vector, *w*, such that:

$$y_{i}=w^{T}s_{i}+\lambda \|w\|$$ (1)

where *λ* is the regularization parameter. A non-zero entry in *w* shows the relevance of its respective PCA component to predicting patients. Then, we found the dominance probability of each PCA component, defined as the number of times it appeared as a non-zero entry in *w* divided by the 1000 times the regression was run. We used sklearn.linear_model.LassoCV (Pedregosa et al., 2011) to fit the model. This function uses kfold cross-validation within the training set to find the best model parameters and the regularization strength. The parameters used for LassoCV were: optimization tolerance of 10^–4^, 3 fold CV, without fitting the intercept of the model, 100,000 maximum iterations, 100 number of lambdas along the regularization path, and the other parameters set to default. Subsequently, we selected the top four components with the highest probabilities, and used those components to train a logistic regression classifier for predicting patients, and to determine the precision and accuracy of classification using these four modes of variation. We used sklearn.linear_model.LogisticRegressionCV (Pedregosa et al., 2011) to fit the classification model. This function also uses k_fold cross validation to find the best model. The parameters used for the function were: lbfgs solver, 100,000 maximum iterations, ROC AUC metric used for selecting the best model, 3 fold CV, and the other parameters set to default values.

#### 2.3.5 Linear Discrimination of Variation

To analyze shape variation between the subgroups of patients with CHF and with pulmonary hypertension, and the distribution of individual shapes among these groups, a linear discrimination of variation was created, as follows: In the group of patients with CHF, the mean shape (*i.e.,* average correspondence particle locations) of the group of patients with CHF but without TR, was compared to the mean shape of the group with CHF and TR. The linear discrimination between the two groups was defined as the difference vector between the two mean shape vectors. The shape of each subject was then mapped/projected onto this vector by taking the dot product between the subject-specific shape representation (the particle correspondences) and this difference vector. This mapping results in a single scalar value (or a “shape-based score”) that places a subject-specific anatomy on a group-based shape difference that is statistically derived from the shape population. For the purpose of interpretability, the mappings of the group mean shapes were normalized to −1 (patients with CHF and TR) and 1 (CHF without TR). The mappings of all the other subjects were then similarly normalized relative to these values, giving a shape distribution of individual members of the population relative to the mean shapes of their respective groups. A univariate Gaussian distribution was then fit to the normalized mapping of each group, to define the probability density function of the shape scores for each group. The same process was repeated for the group of patients with pulmonary hypertension.

#### 2.3.6 Statistical Significance of Shape Differences

To determine which RV correspondence points represented areas of statistically significant differences in shape, a Hotelling t-squared statistic was calculated for each point, and corrected for the false discovery rate; *p* < 0.05 was considered significant (Cates, 2010).

## 3 Results

Of 78 total MRIs meeting criteria for inclusion (see Methods: patient selection), 24 were excluded due to inadequate image quality. The remaining 54 MRIs comprised: 21 patients with TR; 27 comorbidity-matched controls; and 6 healthy volunteers. Table 1 contains patient-level characteristics. Unsurprisingly, end-diastolic and end-systolic RV volumes were significantly higher in patients with TR compared to matched controls, and RV ejection fraction was significantly lower. Ejection fraction could not be calculated for the 7 patients in atrial fibrillation at the time of MRI.

**TABLE 1:** Patient Characteristics. RV = Right ventricle. EF = ejection fraction. EDV = End-Diastolic Volume, EDVI = End-Diastolic Volume Indexed to body surface area, ESV = End-Systolic Volume, ESVI = End-Systolic Volume Indexed to body surface area.

	Disease-Matched Controls	Patients with TR	p
Age	64 ± 10	59 ± 18	
RVEF	49.9 ± 11.2%	42.6 ± 12.8	0.03
RV EDV	150.9 ± 43.5 ml	184.6 ± 65	0.03
RV EDVI	76.4 ± 22.5	105.2 ± 36	0.02
RV ESV	76.6 ± 29.5	106 ± 44.5	0.007
RV ESVI	40.7 ± 15	62.4 ± 28.6	0.002

In the patient group, TR was secondary to pulmonary hypertension in three patients (as documented in the medical record by their treating cardiologist); congestive heart failure (CHF) in 9 patients; and other causes (atrial fibrillation, pacemaker lead injury, congenital heart disease) in 9 patients. In the control group, four patients had a diagnosis of pulmonary hypertension, 12 had CHF, and 11 had other cardiac diagnoses (atrial fibrillation, pacemaker implantation, congenital heart disease). The healthy volunteers had no diagnosis of cardiac disease and no cardiovascular risk factors.

**FIGURE 3:**
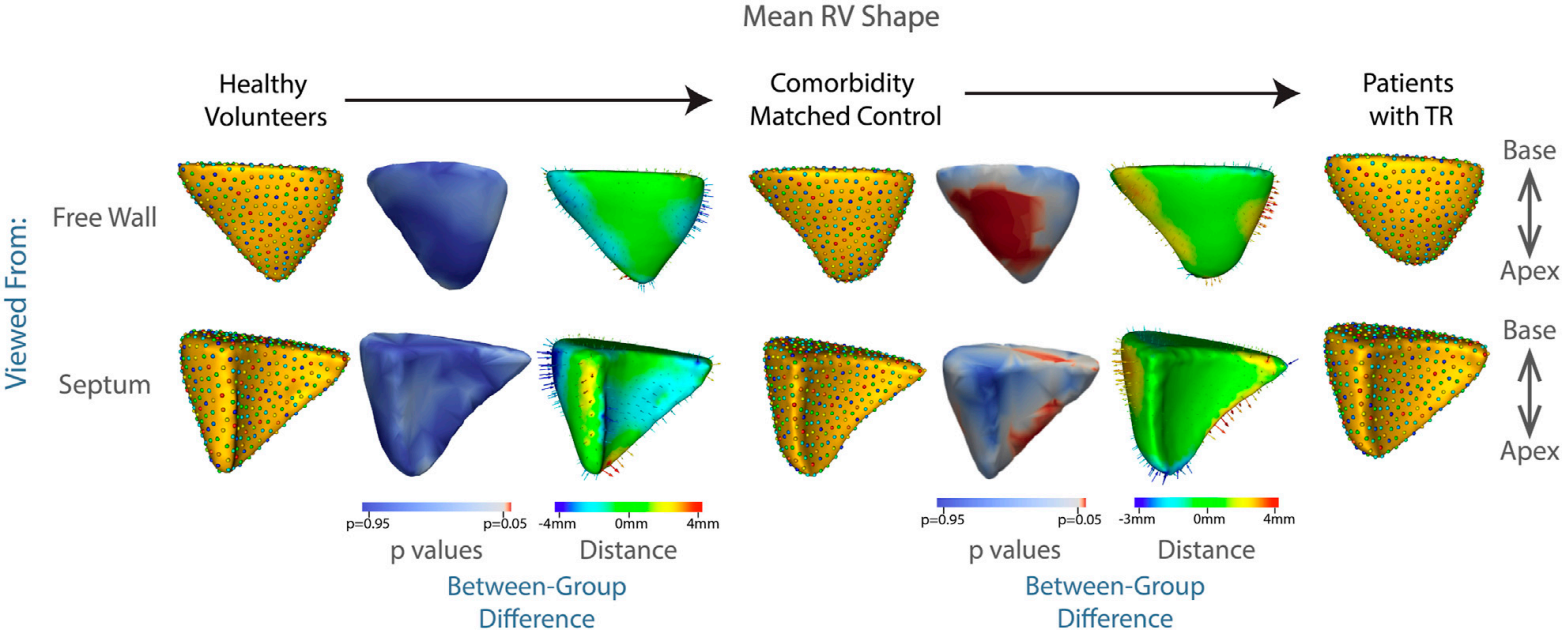
Summary of mean shapes. Columns, 1, 4, and 7 contain the mean shape of the right ventricle in healthy volunteers, patients with cardiac comorbidities, and patients with moderate or greater TR, respectively. Columns 3 and 6 show the difference between the group-mean shapes. The arrows indicate the direction of group differences, and the color represents the magnitude of the group difference. Columns 2 and 5 show the *p*-values of the group differences. The regions with red color showcase statistically significant group differences.

The technique of particle-based shape modeling was then applied to identify differences in group-level RV shape. Figure 3 depicts the mean end-diastolic RV shape of each group, with the regions of between-group difference color-coded on the RV shapes. There is a distinct shape change between the group of healthy volunteers compared to the shape of the comorbidity-matched controls, particularly with inward displacement of the mid-RV free wall. This change reverses as TR develops, with outward protrusion of the RV free wall, widening of the RV base and blunting of the apex. This change is statistically significant, with *p* values approaching zero for the protrusion of the RV free wall, and <0.05 for large regions of the RV (250 of 512 total correspondence points, or 48.8% of the RV surface), as shown in Figure 3.

**FIGURE 4:**
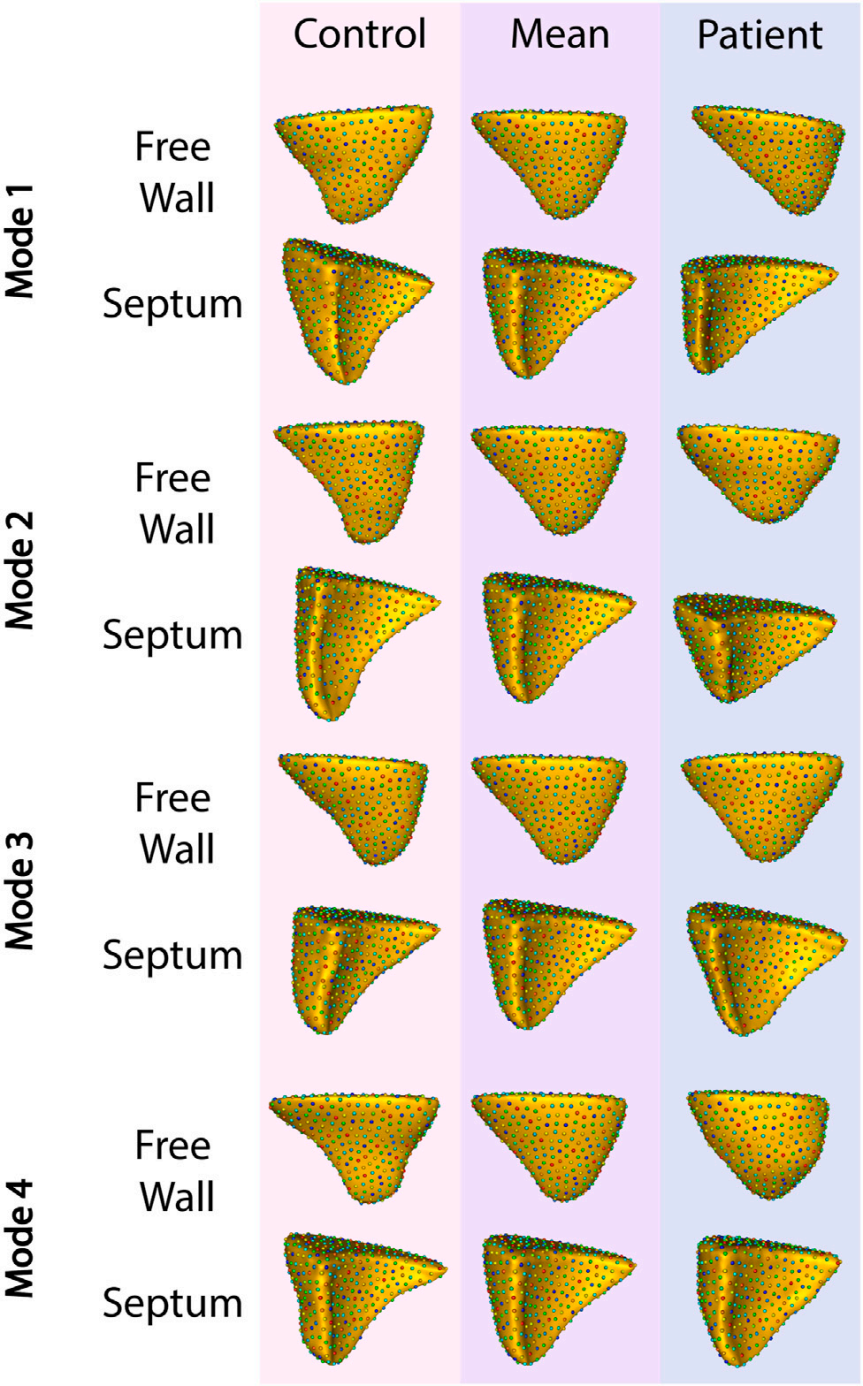
The four primary modes of shape variation between patients with TR and controls. The control group is shown in the leftmost column, patient group in the rightmost, and the overall mean in the center.

To identify granular features of RV shape specific to patients with TR, we divided the overall group of controls (comorbidity-matched and healthy volunteers) and patients with TR into training and testing sets and performed a principal component analysis (PCA). Thirty-one distinct modes of shape variation accounted for 99% of the shape variability between the group with TR and the control group. We identified the four most dominant modes shown in Figure 4.

Applying this analysis to the testing set, these four modes of variation correctly distinguished between RV shapes with TR compared to matched controls with 82% recall, 87% precision, and 82% accuracy. The F1 score for this model was 82%.

We used sklearn.metric (Pedregosa et al., 2011) to calculate all the metrics of classification model. Based on the confusion matrix of Table 2, the formulas for the metrics are:

$$Accuracy=\frac{TN+TP}{TN+TP+FN+FP},Precision=\frac{TP}{TP+FP},$$

$$Recall=\frac{TP}{TP+FN},F1=2\frac{Recall*Precision}{Recall+Precision}$$

**FIGURE 5:**
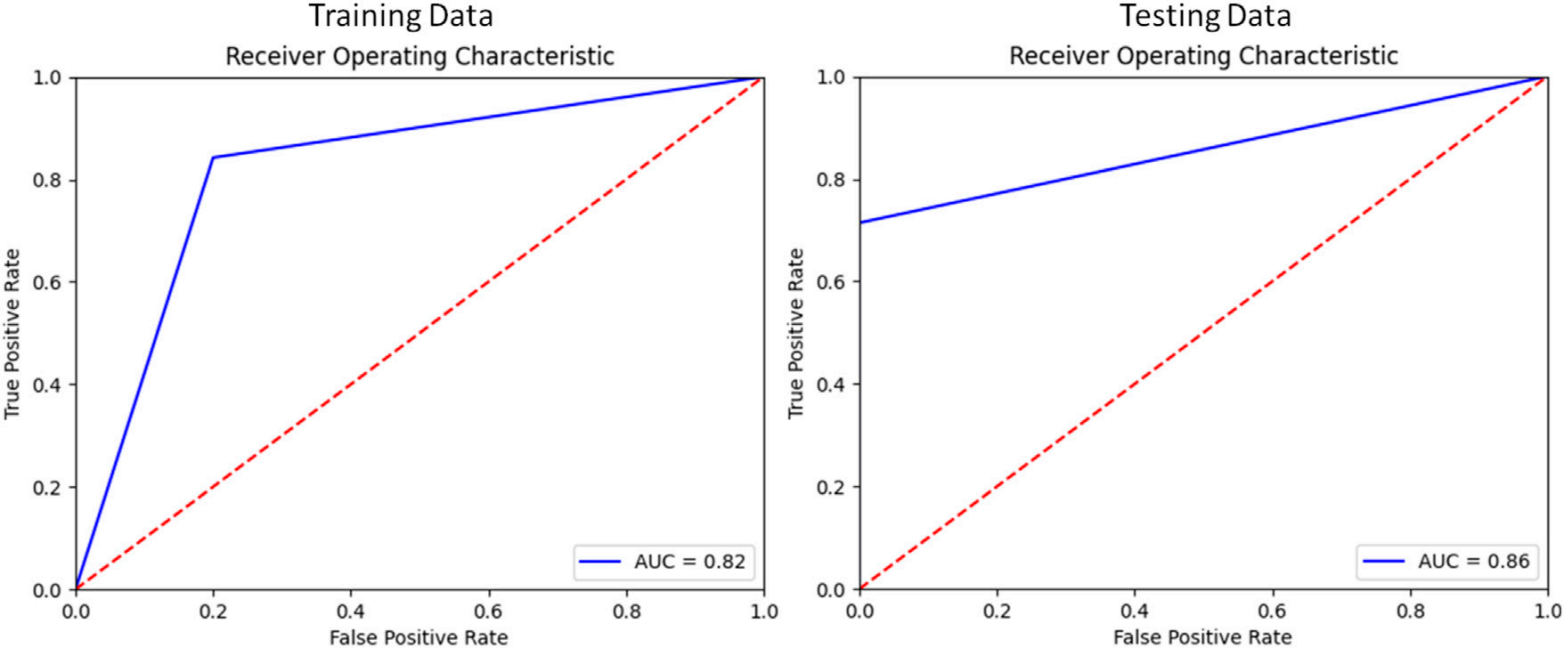
Receiver Operator Characteristic of the Classification model for Training and Testing splits.

Figure 5 contains the ROC curve (receiver operating characteristic) for the model plotted using sklearn.metrics.roc_curve and sklearn.metrics.auc (Pedregosa et al., 2011).

**TABLE 2:** Confusion matrix for Binary classification. TN = True negatives, TP = True Positives, FN = False Negatives, FP = False positives.

		Predicted	
Actual		Negative	Positive
	Negative	TN	FP
	Positive	FN	TP

### 3.1 TR Secondary to Pulmonary Hypertension

**FIGURE 6:**
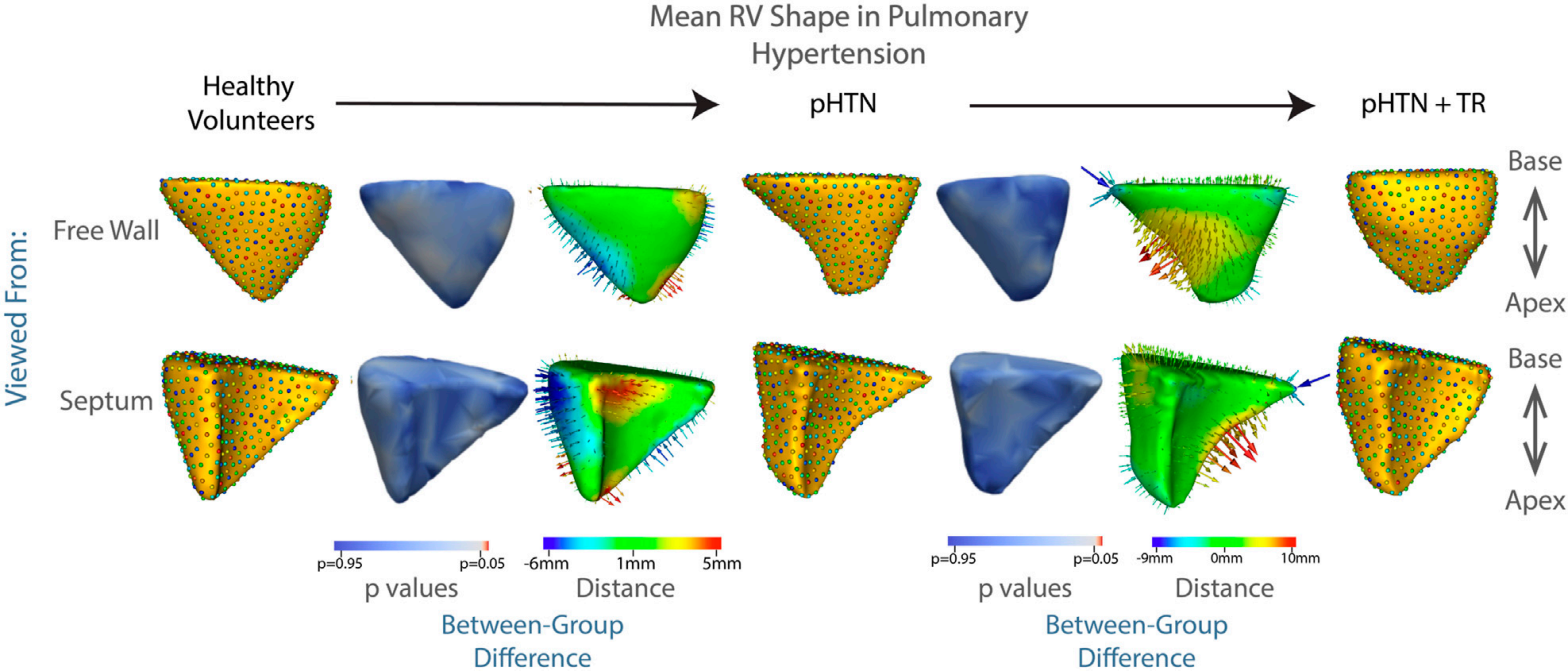
The mean shape of the right ventricle in healthy volunteers, patients with pulmonary hypertension, and patients with pulmonary hypertension plus moderate or greater TR are shown in columns 1,4,7. Columns 3 and 6 show the difference between the group mean shapes. The arrows indicate the direction of group differences, and the color represents the magnitude of the group difference. Columns 2 and 5 show the *p*-values of the group differences. The regions with red color showcase statistically significant group differences.

Of all etiologies of TR, one of the most difficult to treat is pulmonary hypertension. In this disease, RV dilation and resulting TR occur due to narrowing or obstruction of the pulmonary arteries. These arterial changes result in increased resistance to blood flow, as the RV attempts to eject the same amount of blood through a smaller outlet. In the early stages of disease, the RV compensates *via* hypertrophy of muscle fibers and increased expression of cytoskeletal contractile proteins, allowing for increased force generation (Ryan, 2014; Onno, 2015; Thenappan et al., 2016; Prins et al., 2019). However, as the disease progresses, the compensatory mechanisms of the RV ultimately fail. At this point, the RV progressively dilates and the tricuspid leaflets are unable to close, leading to TR. With RV failure and TR comes decreased cardiac output and eventually death. Our results make this trajectory visible—from the shape of the RV in healthy volunteers, to the more streamlined, muscular shape of the RV in patients with pulmonary hypertension but without TR, in whom the RV is well compensated. Following development of TR, the RV balloons outward, indicating progression towards RV failure, shown in Figure 6. These results did not reach statistical significance, likely due to the small sample size, with the lowest *p* value associated with protrusion of the RV free wall at *p* = 0.13.

### 3.2 TR Secondary to Congestive Heart Failure

**FIGURE 7:**
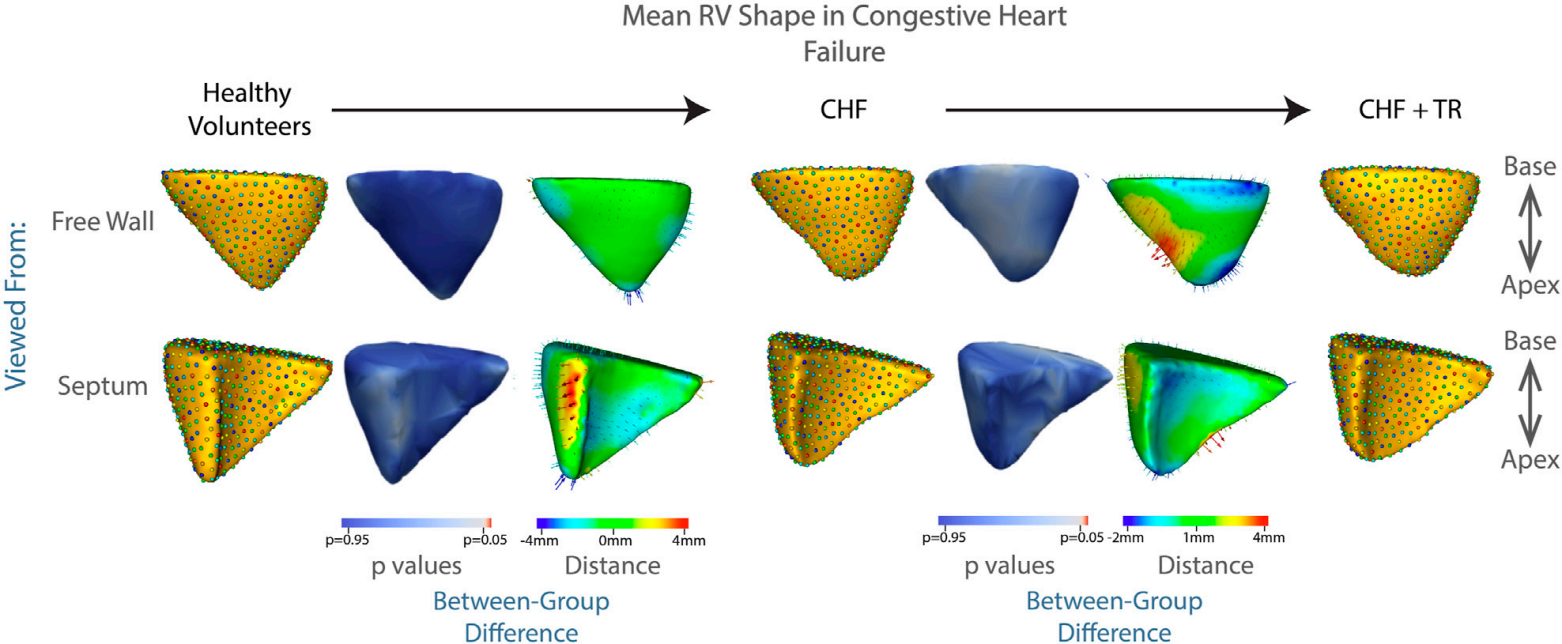
Progression of RV shape change in congestive heart failure (CHF). The mean shape of the right ventricle in healthy volunteers, patients with CHF without TR, and patients with CHF and TR are shown in columns 1, 4, 7. Columns 3 and 6 show the difference between the group mean shapes. The arrows indicate the direction of group differences, and the color represents the magnitude of the group difference. Columns 2 and 5 show the *p*-values of the group differences. The regions with red color showcase statistically significant group differences.

Failure of the heart to supply adequate blood to the body is known as congestive heart failure (CHF). While many of the diseases leading to CHF may affect primarily or initially the left ventricle, there is a more global distribution of disease than in pulmonary hypertension. RV dysfunction can occur due to direct involvement in the underlying disease process, such as ischemia or genetic cardiomyopathy, or as a secondary response to volume overload as the left ventricle fails. As the RV becomes affected by volume overload, it may initially compensate in a way similar to that seen in pulmonary hypertension (if the RV itself is uninjured by the pathologic process causing the CHF) (Voelkel Norbert et al., 2006). However, as compensatory mechanisms fail, the RV becomes increasingly dilated, leading to failure of leaflet coaptation, and resulting in TR. In our patient population, this process is evident in Figure 7. These results did not reach statistical significance, with the lowest *p* value again associated with protrusion of the RV free wall, at *p* = 0.1.

### 3.3 Linear Discrimination of Variation by TR Etiology

**FIGURE 8:**
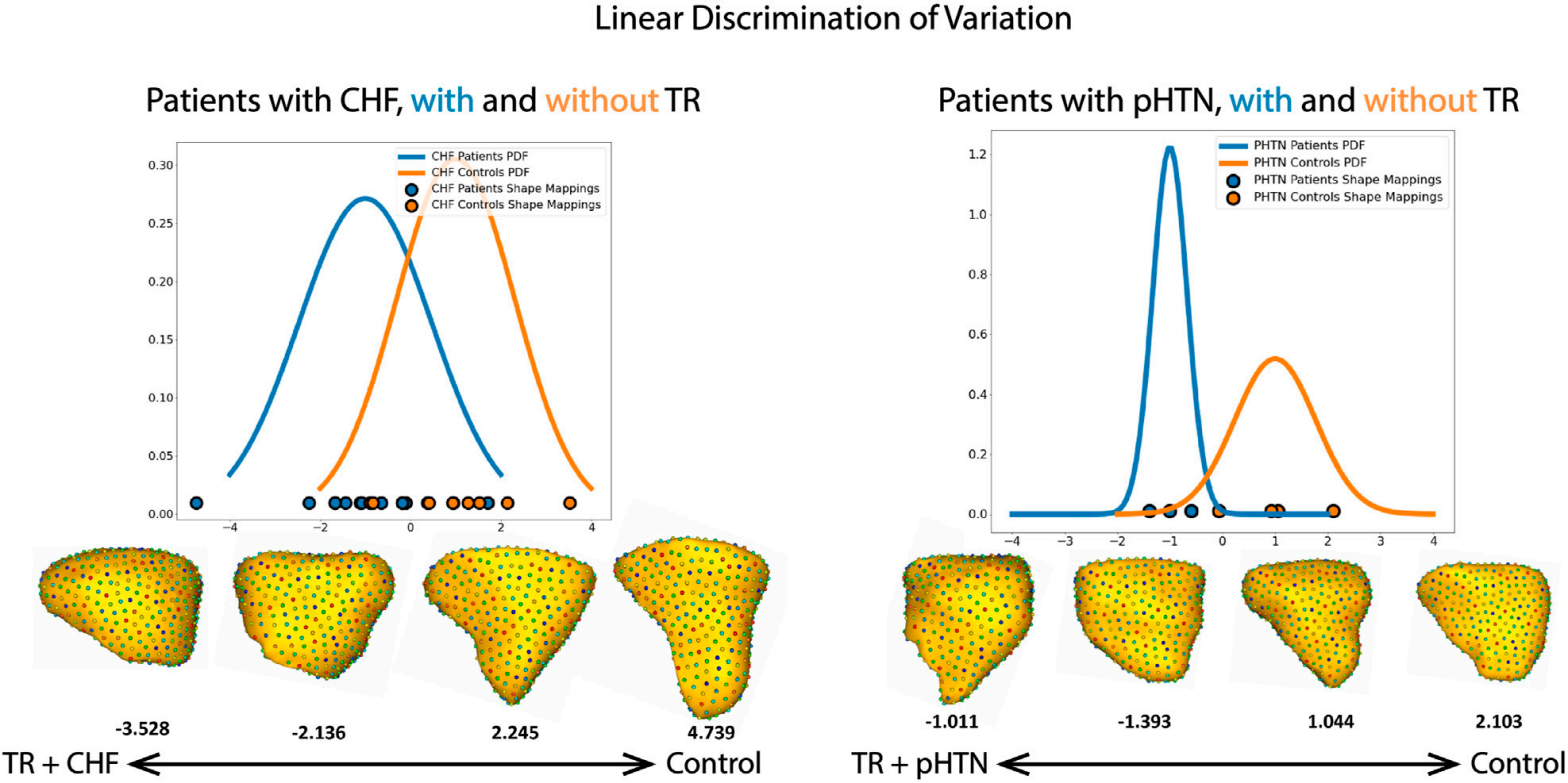
Shape mapping to linear discrimination of variation between population means, for the subgroups of patients with CHF and pulmonary hypertension (pHTN). The group mean for patients with TR is set as −1, and for patients without TR is set as 1. PDF = probability density function. Selected RV shapes correspond to individual points on the graph, and are shown as seen from the free wall. The number below each shape denote the single scalar value (or a “shape-based-score”) that places a subject-specific anatomy on a group-based shape difference that is statistically derived from the shape population.

While common modes of shape variation characterized the RV changes seen in TR regardless of etiology, the model had different abilities to discriminate between patient and control RVs due to the distinct changes seen in pulmonary hypertension compared to CHF. Figure 8 depicts the mapping of each RV shape within these subgroups to a linear discrimination of variation based on population mean shapes (see Methods: Linear Discrimination of Variation). In the group of patients with CHF, 75% of RV shapes fell into an overlapping region of shape described by characteristics of both those with TR and controls. However, in the group of patients with pulmonary hypertension, 57% of RV shapes were clustered in distinct regions on either the TR or control side, with a minority demonstrating shapes that could be characteristic of either the TR or control state.

## 4 Discussion

In this study, we aimed to:

1) Develop a population-level anatomical description of RV shape, directly from detailed 3D models of RVs generated from cardiac MRI
2) Discover statistically significant group differences
3) Classify the RV shape changes characteristic of TR
4) Examine whether those changes are similar in various TR etiologies

### 4.1 Population-Level 3D RV Shape

Constructing 3-dimensional models of 54 RVs, we quantified mean RV shape in healthy controls, patients with cardiac disease, and patients with TR. Highlighting the complexity of RV shape in general, our principal component analysis identified 31 individual modes of shape variation required to explain 99% of the variation between the mean RV shape of patients with TR compared to controls.

### 4.2 RV Shape Differences Between Groups

We demonstrated that the fine details of RV shape in patients with TR are significantly different over large regions (250 out of 512 correspondence points) of the RV when compared to comorbidity-matched controls. This heterogeneity was particularly noted with bulging of the free wall, blunting of the apex, and widening of the RV base (*p* < 0.05 for large regions of the RV, *p* approaching 0 for the protruding RV free wall, see Figure 3). Highlighting the potential for shape modeling as a diagnostic tool in cardiac disease, we trained a linear regression algorithm to identify the RV shapes of those patients with TR versus matched controls, with >80% recall, sensitivity, and specificity.

### 4.3 Characteristic RV Shape Changes

Patients with TR showed a consistent bulging along the free wall of the RV regardless of etiology (Figures 3, 6, 7), consistent with the known underlying RV volume overload occurring in TR (Vargas Abello et al., 2013). Also noted were blunting of the RV apex, and narrowing of the base. These features correspond in granular detail to the known overall transition from a more triangular towards a more spherical shape, as documented in prior echocardiographic studies, as will be discussed below.

### 4.4 Changes Unique to Pulmonary Hypertension and CHF

While our overall subgroup analysis of mean RV shape in patients with CHF and pulmonary hypertension did not reach statistical significance, likely due to the small number of patients in each group, there were striking qualitative differences which correlate well to the known pathophysiologies of each condition, and with distinct relevance to our understanding of RV/pulmonary arterial coupling. First, as seen in Figure 6, for patients with pulmonary hypertension we noted a shape change from healthy volunteer RV shape to the ‘well compensated’ pulmonary hypertension RV shape. These RVs exhibited narrowing of the mid RV and a streamlining of shape, which corresponds well to the known muscular hypertrophy which occurs in these patients as the RV adapts to pump against a higher pressure pulmonary circulation (Prins et al., 2019; Thenappan et al., 2016; Ryan, 2014; Onno, 2015). As coupling ultimately fails, and RV filling becomes volume dependent, we see a decompensated RV shape consistent with the rest of the TR group *i.e.,* with outward bulging of the RV free wall. This shape change is characteristic and was detectable in a computational algorithm; for example, analyzing each 3D RV shape individually using a linear discrimination of variation, the majority of RV shapes in our pulmonary hypertension groups clustered into regions marked by either the TR or control shapes, (Figure 8). Comparatively, the group of patients with CHF showed a 75% overlap in RV shape between the CHF controls and patients with CHF and TR, as shown in Figure 8. This finding is consistent with the differing pathophysiologies of these two disease processes, described above; whereas CHF may affect the right ventricle and tricuspid valve with varying severity and at different time points of disease, in pulmonary hypertension the development of TR is typically a marker of severe disease with associated RV failure (Chen et al., 2018). As such, we posit that it presents with a distinct shape change upon the transition from well-to poorly-compensated.

### 4.5 Conclusion

To date, cardiac shape analysis has focused primarily on the left heart. It has, for example, been used to predict stroke risk based on the shape of the left atrial appendage (Di Biase et al., 2012; Bhalodia et al., 2019). When applied to the more complex RV, shape analysis using echocardiography at end-diastole has demonstrated alterations in gross global parameters including increased bulging of the lateral RV wall from the base to the apex in patients with pulmonary hypertension (Leary et al., 2012). MRI-based shape analysis of cardiac motion, comparing the end-systolic to end-diastolic RV shape, has been shown to correlate with risk of death in patients with pulmonary hypertension (Dawes et al., 2017). To our knowledge there has been no prior analysis of the complex RV shape changes associated with TR.

In our study, across all groups, we observed a progression of RV shape from healthy, to compensating for pulmonary hypertension and CHF, to poorly compensated with TR. The fact that this progression occurs on a spectrum, both in the overall cohort and in the subgroups of patients with the particularly vexing problems of pulmonary hypertension and CHF, favors the idea that the RV shape for a particular patient can be scored along this spectrum, from health to decompensated disease. As such, we posit that this shape analysis pipeline will become the backbone of future diagnostic and prognostic tools. Such a toolkit will aid in identifying those at highest risk of RV dysfunction, guiding therapy and prompting intervention before time is up.

### 4.6 Limitations

This study was obtained using retrospective data. The size of our dataset was limited due to the historically small numbers of patients undergoing cardiac MRI for TR—although this number will continue to increase in the future, as MRI is now part of the standard armamentarium for TR evaluation (Hahn et al., 2019). Consequently, statistical methods were used, as described above, to enhance the size of the dataset and render group sizes approximately equal. Further, invasive hemodynamic measurements (right heart catheterization) were available for very few of the patients included in the study, limiting our ability to compare patient volume status and grade the severity of right heart failure and pulmonary hypertension.

## Data Availability

The raw data supporting the conclusion of this article will be made available by the authors, without undue reservation.
